# Sensitizing the social-ecosystems of outdoor sport environments: A comprehensive framework

**DOI:** 10.3389/fspor.2022.937765

**Published:** 2022-08-16

**Authors:** Sylvia Trendafilova, Vassilios Ziakas

**Affiliations:** ^1^Department of Kinesiology, Recreation and Sport Studies, The University of Tennessee, Knoxville, TN, United States; ^2^Leisure Insights Consultancy, Leeds, United Kingdom

**Keywords:** environment, sport ecology, outdoor sports, sustainability, culture

## Abstract

This paper focuses on the social features of participation in outdoor sports that play a significant role in the lived experience of participants, and in their interactions with the environment. These embodied interactions can bridge nature and culture, and inform interventions for more sustainable ecosystems. Conceptual methods were used to explain the sport-nature-culture nexus and postulate an interdisciplinary framework of social sport ecology, incorporating management, nature sports, neo-tribalism, and non-representation theoretical perspectives. The proposed framework suggests that multi-sensory stimuli, embodied sport practices and neo-tribal cultural values shape the “sports ecosphere,” which needs to be attuned with the affective/cognitive dimensions of experience in ways that build caring cultures for the environment. The significance of this work lies in its comprehensive perspective to the environmental management of outdoor sports by demonstrating the critical role of politics, culture, experience and movement in contemporary sport. It suggests a holistic approach of social sport ecology to better understand and reimagine the environmental practices and character of outdoor sports.

## Introduction

Outdoor sports and the associated activities take place in open areas and in natural settings. In such settings, the social aspects of participation play a significant role not only in the lived experience of the participants, but also in their interactions with the environment. Environmental concerns about outdoor recreational sports are not new. Even though problems such as soil erosion and vegetation decline have become increasingly serious, attention to environmental matters related to outdoor activities dates back to the early part of the twentieth century, as exemplified by the experimental studies of Meinecke (1928) in the United States and Bates (1935, 1938) in the United Kingdom. Both scholars have found negative effects of recreation and nature-based activities on vegetation and soil. Similarly, Vankat and Major (1978) illustrated that the plant species composition in Sequoia National Park in the United States has seriously changed because of increased use of the park. Furthermore, the reindeer density in Finland has been diminished as a result of outdoor recreational activities (Helle and Sarkela, 1993). Similar environmental problems have been reported in Australia, where an increase in participation causes declines in vegetation, erosion, and stress on water resources (Hall, 1994).

Two main factors contribute to the environmental concerns associated with outdoor sports: (1) as the world population is increasing, the number of potential outdoor participants is increasing as well, and (2) the growth in popularity of extreme and alternative sports (Griffin, 2002; Melo et al., 2020a,b). Additionally, manufacturers of sporting goods and the affiliated retailers are seeking to increase the outdoor recreation equipment sales (Ryan, 2004). As a result, the interest in and the demand for outdoor sports has increased considerably, placing even more emphasis on the environmental management of natural resources.

Recent advances on managing the environmental impacts of sport have led to the conceptualization of sport ecology as a subdiscipline of sport management (McCullough et al., 2020). Nevertheless, existent approaches are primarily underpinned by institutional, political economy, ecological and impact assessment frameworks (Trendafilova and Chalip, 2007; Trendafilova and Waller, 2011; Collins et al., 2012; Trendafilova et al., 2013; Collins and Cooper, 2017; McCullough et al., 2018). Quite surprisingly, current sport management perspectives fail to account for how social interchanges mediate the relationship between outdoor sports and the environment, which limits appreciation of the interplay of social dynamics and the natural world. This interplay is what creates *meaning* for participants and drives their behavioral practices. Therefore, it needs to be thoroughly understood before attempting to change behaviors. More generally, sport activities are the embodiment of values, norms and perceptions moderated by the fandom qualities of a sport (reflecting participants' makeup of sport attachment) and its intersubjective atmospheric components (e.g., aesthetics, social interaction, identity, dramatic performance, etc.) experienced within the spaces that performances are unfolded. To capture this embodied meaning in outdoor sports and their connection with the environment, multi-layered insights are needed with new questions emerging. For instance, how does fandom influence behavior of outdoor sport participants and their environmental practices? What social processes do contribute to the shaping of atmospheres in outdoor sport spaces (sportscapes)? How do atmospheres evoke affective responses of participants? These are just some questions that inquiries on the sport ecology of outdoor spaces are worth exploring in order to construct a more sensitized social-ecosystem perspective. Alas, the prevailing institutional logics and economic instrumentalism (Robertson et al., 2021; Chen, 2022; Stenling and Fahlén, 2022), which are principally shaping the sport management discourse, do not seem to be receptive of alternative approaches to measurement, evaluation, policy, and strategizing. They actually constrain the development of transdisciplinary approaches that are necessary to appreciate thoroughly the complex relationship between sport and the environment.

On the other hand, in leisure studies, the concept of “nature sports” has been suggested as a unifying one of the wide range of activities and pursuits enacted in the natural environment (Melo et al., 2020a). This approach by assuming an active role for nature attempts to capture the ways that physical environment in nature sports articulates an alternative ontology in which nature and culture embrace and form “co-constitutive” relationships (Booth, 2020). From this standpoint, it is important to understand the embodied interactions of participants with the surfaces, textures and fluids of physical geographical features as well as the dynamic forces that create them. As such, the elements of natural environments produce affects and sensations that inscribe themselves on bodies and may transform them (Booth, 2020). Centring on these embodied interactions cannot only bridge the divide between nature and culture, but also inform interventions to build more sustainable, social ecosystems of care toward the environment.

In response, we aim to build a comprehensive framework for managing outdoor sportscapes as socio-ecological systems. This frames our approach of a social sport ecology, which is predicated on the thesis that outdoor sport participants develop a reciprocal relationship with the natural environment. Drawing upon Trendafilova and Chalip's (2007) earlier work on the political economy of managing outdoor sport environments, we hereby refine and intensify this analytical lens by integrating two further strands of theory; one is neo-tribalism rooted in sociology (Maffesoli, 1996), and the other is non-representational theory originated in cultural geography (Thrift, 2007). Neo-tribes replace the notion of subcultures as they more accurately capture the changing behaviors and fandom patterns of outdoor sport participants. In addition, non-representational theory with its focus on “*what is felt”* can better explain the phenomenological creation of atmospheres, emotional responses and feelings experienced within natural sportscapes that (re)shape attitudes toward the environment. Taken together, this interdisciplinary perspective provides an integrative analytical lens that sheds light on the dynamics of social-ecosystems embedded into outdoor sport environments.

## Theoretical foundations

We begin with the introduction of Hardin's (1968) classic work on the “tragedy of the commons,” discussing some of its implications in relation to outdoor sports. Next, we present a brief overview of the theory of collective action. We conclude by introducing two new theories: neo-tribalism and non-representational premises, both of which align with the concept of collective action and enhance the understanding of outdoor sport environments.

### The tragedy of the commons

To understand some of the underlying variables associated with the increased demand for outdoor spaces, Hardin's (1968) explanation of the commons problem provides a useful lens through which to better grasp the commons dilemma. Specifically, when participants are provided with common-pool resources, the natural behavior is for those resources to be used to the point of overuse (e.g., surpassing the carrying capacity). This is especially true in cases of public lands with no formal restrictions, leading to depletion and total ruin. Such situations are rather complex, because the individual's negative impact is hardly noticeable, but the benefits to that same individual far outweigh the negative consequences of their own behavior. Scholarship has supported Hardin's dilemma of the commons (Ehrlich and Holdren, 1971; Hardin, 1985; Campbell et al., 2005), and emphasis is now placed on the proactive rather than reactive approach to the management of environmental issues associated with outdoor spaces (Reynolds and Elson, 1996; Sun and Walsh, 1998; Mason and Leberman, 2000; Goeft and Alder, 2001; Lansberg et al., 2001).

Although Hardin (1968, 1985) had suggested that the best and possibly only approach to prevent a tragedy of the commons is to implement practices that are based on sanctions, such approach is yet to be proved successful. Other approaches have focused on technological advances such as artificial landscapes for cross-country skiing and canoe slalom (Backman and Svensson, 2022), and although technical remedies may be necessary, they are not sufficient. Technological solutions are hindered by the fact that land on Earth is a finite resource. Furthermore, any given ecosystem can recover and regenerate, but both processes have limited time to reach the point of return. Lastly, no matter how much technological advance humans are able to achieve, the ecosystem in which we live cannot be replaced. A more sustainable approach would be to foster cooperation and collaboration among users, emphasizing the need to integrate social and political theory with biological metrics. Therefore, based on the fact that the tragedy of the commons has its roots in social behavior, efforts for long-term solutions need to be founded in the social sciences, and more specifically in strategies to modify human behavior. In particular, Ostrom's (2000) work explored why some groups self-organize and others do not, emphasizing the relevance of the theory of collective action to the governance and management of natural resources. In the search for solutions to modify human behaviors, the effort must be focused on increasing the authority of individuals to devise their own rules. This, in turn, has the potential to create social norms that evolve and increase the probability of individuals better solving collective action problems (Ostrom, 2000). In other words, ordinary people are capable of creating rules and institutions that allow for the sustainable and equitable management of shared natural resources.

### Collective action

It is clear that failed collective action is the main source for the tragedy of the commons not to occur. Logically, this translates to addressing the problem by developing effective collective action (Lubell, 2002). In doing so, it is imperative that the collective action provides for means to monitor and encourage environmentally friendly behaviors, based on self-imposed solutions. The work of Olson (1965), however, challenges the possibility of achieving collective action by pointing out the challenge of not only successfully nurturing collective action, but also to the challenge of sustaining it over time. The rationale for such skepticism comes from the possibility of having too many individuals who are free-riders. In other words, the cost associated with collective action is placed upon those who make the effort, but for those who do not, they are still able to enjoy and consume the benefits. This scenario is especially counterproductive as the size of the group increases, thus making the non-collaborators more difficult to identify, leading to the reality that most collective action groups will either not form or not endure.

The main consequence of failed collective action is that for any given situation the socially desirable goals would not be achieved. This is especially critical in the environmental management of outdoor spaces where the key is to find a balance between the individual participant's interests and those of the group (Espejo and Stewart, 1998). The particular challenge is to identify and unite a sufficient number of participants (i.e., critical mass) through a social mechanism based on shared interests, values, and resources (e.g., neo-tribes). As critical mass theory suggests, having unbalanced group dynamics impairs innovative decision-making due to the fact that social pressures encourage minority group members to adopt or conform to the majority's opinions (Velte and Nuber, 2021), therefore the need to form groups with similar values and beliefs. In order to achieve a successful outcome, it is critical that communication is open, objectives are shared by all members of the group, and there are minimal incentives to free-ride (Sally, 1995). Furthermore, it is imperative that communication is face-to-face (Cardenas, 2000). However, the nature of outdoor recreational activities can impede the communication process (essential for collective action) since some of those activities are rather individualistic and/or take place in remote and dispersed natural settings. For this reason, opportunities for formal and informal gatherings, celebrations and/or public events must be put in place so as to encourage group members to talk and share in these collective settings.

In theory, it is possible to achieve collective action if the group size is large enough to encourage cooperation rather than free-riding (Heckathorn, 1996). The critical component is the presence of information that is easily shared among the group members, which in turn would make it difficult for the free-riders to exist and not comply with the group decisions and actions. Therefore, the challenge is to find the optimal group size where cooperation comes naturally and free-riders are easily identified. This is especially important in outdoor recreational settings as each free-rider becomes a source of environmental damage. With the steady increase in the number of outdoor participants, conditions would be favorable for free-riding. Consequently, greater and more persistent levels of collective action are needed to address effectively the problem of free-riding.

In summary, addressing the environmental management of outdoor recreational activities through voluntary collective action is a rather complex endeavor. The spaces in which these activities take place and the social conditions of making it easy to become a free-rider, reduce the likelihood that groups will form, maintain themselves, or be effective. The traditional management approaches have been based on solutions via behavioral restrictions, access fees, and sanctions, without much success in achieving long-term sustainability (Ostrom, 2009; Haddock and Quinn, 2015). Therefore, we suggest the consideration of looking into neo-tribes and their potential for addressing some of these issues.

### Neo-tribes and sport

Membership of participants may span across different fan (or brand) communities as a result of the commoditized over-supply of leisure pursuits, media devices, cultural symbols, and celebrity idols within a conspicuous consumerist society. In this context, fans get self-organized in rather heterogeneous and fluid groups of neo-tribes, which are confined by similar interests, analogous lifestyles, common rituals, and shared language (Maffesoli, 1996). The staging of outdoor sports as spatial-temporal and transient activities provides a rich terrain to tailor themed experiences designed to positively alter the neo-tribal idiosyncrasies of outdoor participants toward the environment.

Contemporary fandom-related behavior and practices in sport can be substantially illumined by neo-tribal theory (Maffesoli, 1996). Neo-tribes are fluid concentrations of fans with unsolidified confines and fluctuating memberships (Bennett, 1999) who share common pursuits, lifestyles, and modes of expression (Hardy et al., 2013). Neo-tribalism maintains that contemporary identities are unstable and disjointed since they are constructed around commercial products, images, and texts as a consequence of taste, aesthetics, and the feelings stirred through engaging in a common activity (Bennett, 1999). For this reason, neo-tribes like better to be consolidated around brands and commercial goods (Cova and Cova, 2002), while they exhibit less deep lines of detachment and more ephemeral associations than subcultures (Bennett, 1999). In this respect, they compose fluctuating groupings with followers stepping from one group to another and being members of multiple tribes. Thus, participants may be members of several outdoor sports. As Maffesoli (1996) argued, members of neo-tribes are based on a mindset and lifestyle that epitomizes a common sense of community and shared taste, feelings, habits, and consumption patterns. Considering the communal and transient character of neo-tribal behavior, it seems that sport provides a suitable platform for temporary gatherings to take place whereby fans enact a common sense of community and parade their identity. The function of sport events as symbolic social spaces (Ziakas and Costa, 2012; Ziakas, 2016) concurs with the need of neo-tribes to voice in public their mindsets by participating in communal pursuits and activities. Subsequently, neo-tribal ties and relationships embody interconnections of inter-group commonalities and intra-group affiliations in sport participation settings.

This performative enactment of sport as a lifestyle by a neo-tribe is similar to communitas, which was defined by Turner (1974) as the temporary antistructure created in public events wherein everyday boundaries, statuses, and ranks cease to apply and participants feel as equals. Moreover, neo-tribes by performing their own aesthetic ethic seek to create moments in which to live out their own values and create temporary pockets of sovereignty over their own existence that exemplify sociality, solidarity, hedonism and vitality (Riley et al., 2010). Thus, neo-tribal fans are inclined to form ephemeral spaces in which to perform a set of shared practices that frame a common bond providing a series of social, hedonistic gatherings that celebrate their identity and belongingness. Sport may accentuate the effects of these processes as it prompts intense attachment to rules, traditions and idealized or “sacred” settings, where they can develop stronger social bonds and affinities. However, neo-tribalism is not without limitations as it does not explain phenomena like localism in/through sport, which may reinforce conservative communitarian tendencies (Towner and Lemarié, 2020). Also, although participants share common interests in an outdoor activity, this does not necessarily mean that they share a common lifestyle. For this reason, attention is needed on the social mechanisms that reinforce conviviality and bonding, even transient, hence forming neo-tribes.

Sport is a social-cultural phenomenon where both participants and fans are connected through shared values, norms, and beliefs. Additionally, sport experiences define and shape not only personal identity, but group membership as well (Kramer and Brewer, 1984; Brewer and Kramer, 1986; Kollock, 1998). In turn, this provides for the potential to leverage group membership as the means to achieve collective action by instilling environmentally-oriented behavioral norms. Although social strategies have been considered as solutions (Poole and Van de Ven, 2004; Andreasen, 2006), the application of social technologies remains to be explored. This is especially true as the wide diffusion of digital technologies is a key mechanism that needs to be leveraged with neo-tribes since they tend to interact through social media.

### Non-representational theory

Rather than examining and representing social relationships and their outcomes, the focus of non-representational theory is on embodied practices. Attention is drawn not on the discourse and what is being said, but on the analysis of what/how is enacted, practiced, embodied and felt. This involves how human and non-human connections, affinities and bonds are enacted or performed to shape embodied configurations of lived experience. More specifically, this perspective aims to capture not what is represented or conveyed, but the social dynamics as performed by human and non-human interactions, the material and non-material world. Therefore, the matter is not simply on what is produced, but instead attention is prioritized on those processes that operate before deliberate, reflective thought and drive human behavior (Thrift, 2007). Affect, atmosphere, hope and politics are key intertwined, performative loci of non-representational theory. This is pivotal for understanding how the relationship of sport participants to the natural environment is shaped, altered, resisted or contested, as this is influenced by tangible and intangible elements of sport, the space in which it is performed, and its underlying socio-political dynamics that configure sportscapes. Non-representational theory has been applied to a wide range of contexts such as dance, music, cinema, walking, gardening, and children's play to name a few (e.g., Thrift, 1997; Crouch, 2003; Harker, 2005; Morton, 2005; Wylie, 2005; Tzanelli, 2019). Nevertheless, applications to sport management are still lacking. This omission constrains the understanding of various affective manifestations of sport in a comprehensive and multidimensional manner. In addition, non-representational tenets can illumine processes and meaning structures of embodied sport practices so as to tailor targeted interventions that link substantively nature, culture and sport. Furthermore, these tenets can play a critical role in coordinating management exigencies under a phenomenological—social sport ecology framework.

In particular, sporting activities are marked by sensuous experiences (Thorpe and Rinehart, 2010) that enable participants to reconstruct the material worlds (Turner, 2008). The idea of nature sports as an interaction between performing bodies and objective features and forces in the physical environment coincides with non-representational thinking shedding light on how these interactions produce affects and sensations that inscribe themselves on, and transform and produce, bodies (Booth, 2020). Understanding interactions between performing bodies and the physical environment brings to the fore the intimate relationships among embodiment, culture, and nature that form sport practices. This line of thinking rejects viewing the world as a set of separate pre-given forms that come together, but instead, treats the world as a dynamic terrain encompassing flows, movements and linkages within which realities are constantly (re)constituted (Anderson, 2012). In this vein, non-representational theory may help us examine how nature sports enable moving and performing bodies to converge with non-human material entities in a process of “mutual becoming” (Booth, 2020). This perspective shifts thinking away from fixed and stable entities toward interconnected flows, mutual interferences and constellations of non-humans, humans and places that “continually merge and emerge” (Anderson, 2012). Otherwise put, embodied sport practices enacted in the environment (re)configure relationships of participants to the social and ecological systems in which they are embedded, therefore, interventions are needed to positively influence the web of interactions among material and non-material elements shaping feelings, emotions and affect.

It should be also noted that non-representational theory considers the emotional, sensual and aesthetic sense of embodiment in regards to its political value. Two notions are pertinent for reimagining the ecology of outdoor sports: the politics of affect and the politics of hope. The politics of affect emphasize the extensive use of emotional techniques in a highly mediatised society to control and manipulate people for political and commercial gains. Obviously, greenwashing is the most common example within the sport industry. New technologies and media promotion mixes are often put in place to elicit affective responses and conceal detrimental environmental practices and impacts. It is important here to identify the manipulative techniques and learn how to manage them. The politics of hope describe the emergence of new forms of counter-politics that propose alternative modes of thinking and being. For instance, eco-friendly practices, eco-aesthetics and cultures of care are alternative models for the sustainable operation and management of outdoor sports. The challenge here is to translate such models into realistic action within the substantive context of outdoor sports (including current practices, values, worldviews, etc.) by crafting and implementing sustainable management strategies that alter harmful environmental behaviors.

## Socio-ecological framework: toward a social sport ecology

Systems logics guided theory-building of the conceptual research undertaken with an aim to delineate inputs and outputs, principal components and their interactions, structural patterns and processes in a comprehensive conceptual framework. The analysis was initially based on Ostrom's (2000) sustainability framework of complex social-ecological systems, which shows the interactions and outcomes among four core subsystems that affect each other as well as link social, economic, and political settings and related ecosystems. The core subsystems are: (i) resource systems (e.g., a protected park); (ii) resource units (e.g., trees, shrubs, and plants, etc.); (iii) governance systems; and (iv) users. Each subsystem consists of multiple second-level variables (e.g., size of a resource system, mobility of a resource unit, level of governance, users' knowledge of the resource system), and is determined by social interactions such as information-sharing among users or networking activities. Therefore, this framework provides a comprehensive set of variables and factors that may affect the relationship between outdoor sports and the environment. By extrapolation, the ensuing conceptual analysis focused on social aspects and interactions as these were shown to be central in shaping attitudes, behaviors and cultures about outdoor sports and the environment, but have not been sufficiently addressed in extant sport ecology scholarship.

The focus on description and explanation of conceptual research leads to a better balance between theory-building and theory-testing research (Meredith, 1993). A combination of conceptual induction and conceptual deduction was used. With conceptual induction, the purpose is to explain a phenomenon through the relationships observed between the system's elements. In other words, the goal is not only to describe the phenomenon accurately, but also to explain how it occurs (Meredith, 1993). Then, through conceptual deduction, a framework is postulated and its ramifications are detailed for comparison with reality, as well as to provide guidelines for managers (Meredith, 1993). Accordingly, we sought to describe and explain the spatial and social configuration of the dynamics that epitomize the sport-nature-culture nexus within the context of outdoor recreation. Central to this inquiry was the integration of neo-tribal and non-representational theories with the management pragmatism shaping outdoor sports. This was deemed necessary not only to portray and expound the multidimensional relationships involved in outdoor sport environments, but also to enable potentials for reimagining alternative realities. More specifically, this concerns the construction of counter-worlds that amplify the environmental and social problems in which we are to stimulate our imagination (Tzanelli, 2020a). In this regard, outdoor sports can be seen as a plural space where participants make sense of reality on their own terms by turning sport practices into a world they can share with others. The generated framework converged the sources of outdoor sports' connection with the environment and the attendant processes leading to caring cultures, which were contrasted with realistic scenarios of environmental action based on authors' experience and long-term involvement with outdoor sports.

**Figure 1:**
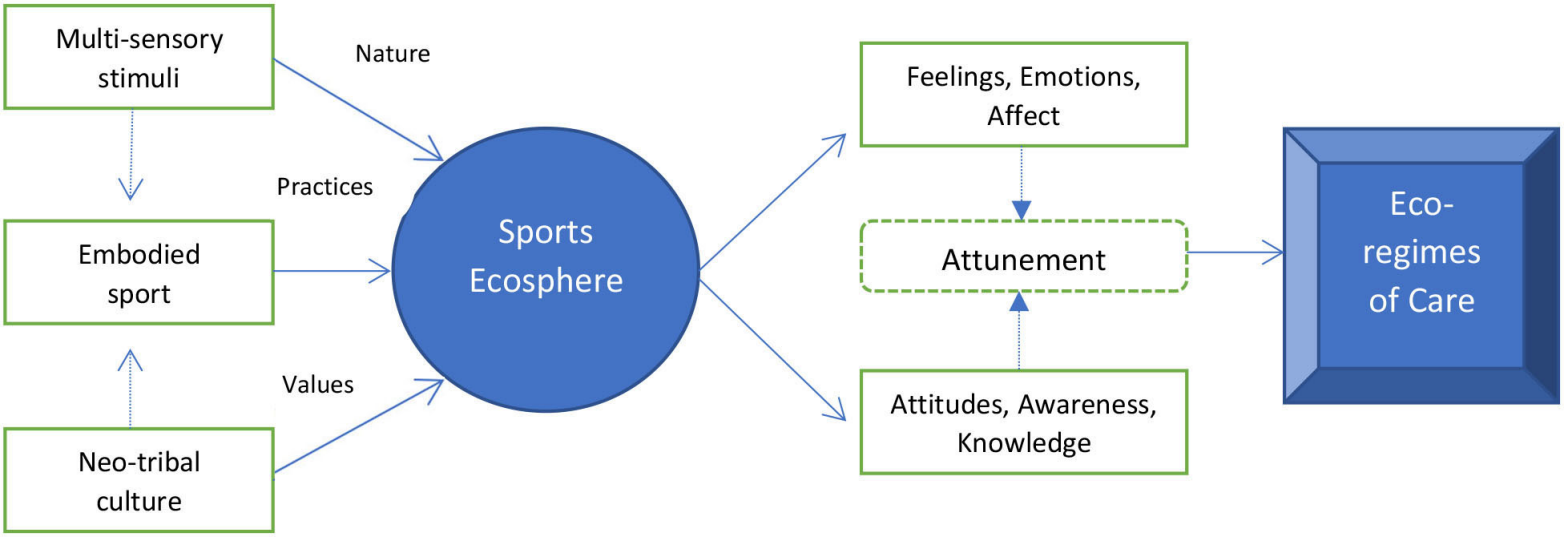
Socio-ecological framework.

In this section, we present a visual illustration of the proposed theoretical framework (Figure 1) and provide a description of its components, including the interplay among them.

As indicated in our framework, the combination of “multi-sensory stimuli” and “neo-tribal culture” affects how sport and the related experience is created, and eventually embodied. This relationship is rather complex as multi-sensory stimuli, through the direct interactions and experiences with nature and the surrounding environment, shape the “sports ecosphere,” defined herein as the socio-ecological system of a particular sport, including ingrained networks of common understanding, activity and socio-spatial organization. Accordingly, the values of a neo-tribal culture influence the level of commitment and desire to not only belong to the sports ecosphere, but also to preserve it and minimize the negative impact on the environment. In other words, neo-tribal values play a key role in the bidirectional relationship between outdoor sport participation and the environment. It is important to note here that the lived experiences within the sports ecosphere, in turn, determine how attitudes, awareness and knowledge develop and evolve over time. The focus needs to remain on increasing awareness and knowledge about the interplay between sport and the environment, and how these two notions affect attitudes. In general, attitudes are situationally specific and the level of awareness and knowledge could negatively or positively impact the emotional sensing of outdoor sport participants. However, simply fostering awareness of environmental issues although necessary, it is not sufficient to generate environmentally conscious behaviors.

If the focus shifts to include neo-tribal values and their potential to address environmental concerns, it is critical to ensure that those using a common outdoor space share similar values. As such, the cultivation of shared values may drive joint behaviors and practices, while fostering trust and mutuality among participants. Neo-tribes represent more feasible action groups than sport subcultures, especially if the ultimate goal is to achieve long-term solutions, because they address fluid lifestyle patterns, routines, and behaviors. The matter then is to capture how they evolve and change over time in order to adapt tailored interventions. Such an adaptive and continuing approach requires that definable neo-tribes among outdoor recreationists be identified and analyzed with reference to their values and practices. This would establish a strong foundation for implementing visionary interventions.

The lived experiences of outdoor participants within the sports ecosphere generate psychological responses expressed in feelings, emotions, and affects. Here the creation of atmospheres can make strong connections between sport practices and environmental integrity. Atmospheres are the effects of connection between humans and places characterized by affective and sensory dimensions (Tzanelli, 2017, 2020b). Outdoor sports by inducing participants' engagement with nature and its animate flora and faunal worlds (Tzanelli, 2020a) may produce atmospheres of environmental consciousness and sensitivity. For example, it is found that free-divers' connection to the underwater world defines their joint actions and identities, which are based on the principles of slow consumption, slow tourism and eco-aesthetic norms and values (Ziakas et al., 2022a). Neo-tribalism then informs that community bonding does not take place only between free-divers themselves, but is consolidated at the intersection between their communications and their individual engagement with the natural world as a world of wonder (Tzanelli, 2020a; Ziakas et al., 2022b). This is a magical-realist world that amplifies reality to such an extent, that the free-diver experiences a spiritual communion with the sea (Tzanelli, 2020a,b). It is in this kind of embodied practice and atmospheric connection to nature that outdoor sports have the potential to create and cultivate neo-tribal communities and cultures of care.

Similarly, Eider et al. (2021) conducted a social-ecological analysis of SCUBA tourism human-environmental interactions and concluded that a densely interconnected network of social cooperation existed. More specifically, human-reef interactions were influenced by the self-organization of SCUBA businesses in the local community. Such interactions have the potential to develop social capital and facilitate conservation efforts. Another illustrative example is the sport of disc golf, which takes place outdoors and in natural settings. Disc golf players express a strong identity with the sport (Trendafilova, 2011), and the socio-ecological framework provides a lens through which their behaviors can be better understood and managed. Therefore, neo-tribes in general, and the neo-tribe of disc golf in particular, can become new sources of identity and subsequently leverage. Behavioral interventions should focus on the identity, lifestyle, values and beliefs of the neo-tribe as they co-constitute the sports ecosphere in which perception, imagination, understanding and emotion combine into embodied experiences. A similar situation is present in the neo-tribe of recreational vehicles' users (RV-ers) where members live in recreational vehicles as an alternative lifestyle through which strong social bonds and sense of community are created (Counts and Counts, 1992).

The proposed framework illustrates a dual pathway for the sports ecosphere to nurture eco-regimes of care toward the environment. One is cognitive and conative, involving attitudes, awareness, and knowledge. The other is affective, encompassing feelings, emotions, and affects. Both interact and have impacts on participants invariably according to the context and the specific sport. For this reason, we argue that the focus should not be on each path leading to an eco-regime of care, but that it rather be on the “attunement” of these two pathways. By attunement we mean the harmonization of cognitive, kinesthetic and emotional sensing of the connectedness between sport and the environment. We posit that this congruency may go beyond empathy to create an experience of reciprocal connectedness with the environment. Therefore, it is critical to learn how to attune the sports ecosphere with the affective and cognitive dimensions of experience in ways that build caring cultures for the environment. This warrants ample room for future research to envision and craft environmental strategies for outdoor sports. The socio-ecological framework provides a firm foundation to do that. Its interdisciplinary nature may encompass mixed quantitative and qualitative research methods to comprehensively capture the interconnectedness of outdoor sports and the environment.

## Management implications

Finding the appropriate interventions for the environmental management of outdoor recreational spaces is not a simple and easy task. Strategies such as to designate natural protected areas have failed to address the underlying commons problem, because they merely shift the demand to unprotected areas (Carrus et al., 2005). Adopting rules and regulations to reduce the possibility of commons tragedy have not been successful either. This is due to the high cost associated with monitoring, especially in natural settings that cover large areas. Furthermore, different political systems (e.g., democratic) may make it unfeasible to restrict usage, even if there are concerns about negative impact on the environment. Worse still, new legislations and regulations usually take time to be ratified and put in place. In contrast, environmental degradation caused by increasing demand in outdoor sports spirals rapidly at a concerning rate. As a result, regulation of environmental practices through laws and rules lag behind the demand and growth of outdoor sports.

The environmental management of outdoor recreational activities is not limited to the local community, it is a global concern that would require strategies and tactics appropriate at the global scale, but at the same time flexible enough to be applied to the local ecosystem. In the design of these strategies, it is imperative that local and regional customs, values and practices are taken into consideration if the goal is to achieve a long-term sustainability. Implementing regulations may serve as a short-term solution, but they will not reverse the increased stress on the natural environment, only temporarily slow down the degradation. Instead, the focus should be on targeting interventions at sport neo-tribes in their regional and local contexts, as effective collective action could be achieved in these social settings. This is especially promising in neo-tribes with shared norms and values, where formal regulations may be unnecessary. Members of those groups can design their own rules, enforce and evaluate them to ensure that if necessary better rules are designed. Behavioral changes will not occur fast, but they will be incremental in nature until the desired behaviors become the norm.

In order to design and implement effective interventions, sport neo-tribes in outdoor recreation must be better understood. Neo-tribes are brought together to share a lifestyle expressed by particular sports, which enables them to adapt to the ever-changing world. The emergence of digital social media facilitates the formation of neo-tribes in online communities that may also keep relationships at-a-distance (Ziakas and Costa, 2015; Lundberg and Ziakas, 2018). Research has also explored the dynamic and changing relationships between emerging digital platforms and digital natives (Nash et al., 2018). For example, digital nomads are a population of individuals where the boundaries between work and leisure are blurred and the advancement of social media provides these individuals with the freedom to easily and frequently relocate (Reichenberger, 2018). Members may belong to multiple neo-tribes, allowing, hence, for more fluid identities and different social circles to interact. This means that roles and identities are not static as assumed in subcultures, but changing and interconnected with multiple lifestyle sports. Thus, interventions may need to be carefully planned by not targeting individual sports separately, but perhaps focusing on a collection of allied outdoor sports whereby neo-tribes take part. Simply put, we need multi-sport interventions coordinated under a joint framework to reach participants as much physically and digitally across their involvement in several neo-tribal outdoor sport communities. Evaluation of interventions based on that perspective can test neo-tribes in sport and the relations between neo-tribes and environmental consciousness, and what is learned in the sport context may have value in other settings. Thus, the effort to explore neo-tribes has both practical and scientific value.

Additionally, non-representational theory provides a unique lens through which to understand and develop solutions to the environmental management of outdoor spaces, especially those associated with alternative sports (Thorpe and Rinehart, 2010). Its strength is in providing a direction for outdoor recreation scholars and capacity to inform research paths into the dynamics of human-nature connections. For example, effect-oriented inquiries can be carried out where different ways of thinking emerge from relations with nature (Douglas, 2020). Outdoor sports (in general) often involve a special relationship with the surrounding environment and the reconceptualisation or reconfiguration of space. In this sense, when considering solutions, it is critical to focus on “things, space, time and nature,” one of the tenets of non-representational theory. An integral part of meaning-making and strategy-making is political determining the use of space, sport practices and synergy (or lack thereof) with the environment. Another important factor to consider is that in outdoor sports and recreational spaces, the environmental conditions are constantly changing and evolving, when compared to sports taking place indoors. This, in turn, presents more challenges and potentially rather complex solutions, requiring a holistic approach. Such approach fits well into the aesthetic lens of non-representational theory due to its life-affirming ethos revealing conviviality, corporeal dynamics and life-bonding features of outdoor sports that have the potential to connect deeply participants with the environment (Larsen, 2022). The proposed socio-ecological framework provides for a comprehensive approach to the environmental management of outdoor sports as it demonstrates the critical role of understanding politics, culture, experience, and movement in contemporary sport.

## Conclusion and future research

In this paper, starting from the thesis that outdoor sports are closely related to nature and, in particular, the environments in which they take place, we offer a comprehensive interdisciplinary framework that can ground future inquiries on making outdoor sports more sustainable. The framework is a synthesis of management, nature sports, neo-tribalism, and non-representation theoretical perspectives. Therefore, it advances the discourse on sport ecology by building a sensitized social-ecosystems approach of outdoor sport environments. With this approach we identify loci and foci for tailored interventions. Our approach is based on the assumption that outdoor sports' participants develop an intimate and reciprocal relationship with the natural world (Brymer and Gray, 2010). Despite the risk that deleterious environmental impacts may occur as a by-product of these activities, such as noise and visual pollution, soil erosion, water and air pollution, natural landscape destruction, fauna and flora destruction, outdoor sports have the potential to address environmental conservation and protection when developed and managed in a sustainable manner (Melo and Gomes, 2016). Feelings connected to nature may foster a desire to care for the natural world and contribute to more environmentally sustainable practices (Brymer et al., 2009). The key is to develop more sensitized strategies and tools for doing that by fostering sports' communion with nature (Howe and Morris, 2009; Atkinson, 2010; Howe, 2016).

Trends indicate that the growth of outdoor sports continues with participation being widespread around the globe, outpacing the increase of most traditional sports in many Western nations (Wheaton, 2013; Brymer and Schweitzer, 2017). With the proposed socio-ecological framework we challenge scholars to think holistically and creatively in order to balance participation with environmental sustainability, as well as to consider how the neo-tribal and embodied practices of outdoor sports may result in new forms and configurations congruent to environmental protection. These configurations will probably be pertinent to practices that seek to preserve and foster a harmony with the natural world. Sensitizing configurations of the complex sport-nature-culture entanglements dictates both a conceptual theorization and a practical approach for managing the multidimensional tangible and intangible elements of the world and their relationships that co-constitute the social-ecosystems of outdoor sport environments.

A limitation of the socio-ecological framework is its phenomenological grounding with a focus on the embodiment of practices, which valorizes subjective experiences of the interplay between outdoor sports and nature. While this may limit objectivity of research accounts, the framework is flexible enough to encompass alternative epistemologies cutting across positivist and interpretivist paradigms. The concepts of sports ecosphere and attunement allow (and, if not, necessitate) the application of multiple perspectives and methods to thoroughly capture co-constitutive relationships among nature, culture, and behavior of participants enacted and evolved within intersubjective sportscapes. For example, complex adaptive systems, actor-network theory or netnography can be used in conjunction with more traditional qualitative/quantitative research methods. The goal is to obtain balanced accounts of representational viewpoints (analysis of interviews, text and discourse), non-representational aspects (analysis of embodied practices, atmospheres and affects), as well as quantifiable patterns (analysis of physical stimuli, attitudes and psychological responses), and particularly, their interplay in the shaping of outdoor sportscapes. Such a holistic research approach can enable methodological triangulation and sophistication for producing well-rounded analyses that preclude possible bias or preconceptions. The resonance of this highly heterogeneous angle is vital for the emerging field of sport ecology and the pressing need to envisage alternative realities and models of sustainable sport and leisure management. For this reason, we encourage scholars to utilize mixed methods to analytically explain the constitution of a sports ecosphere and identify pathways leading to eco-regimes of care. This also highlights the main areas for research that the socio-ecological framework suggests for understanding processes underlying the construction of a sports ecosphere, its attunement with environmental protection, and the subsequent cultivation of cultures of care. In this agenda, the following research questions are key:

What is the makeup of a sports ecosphere as shaped by the interplay of multi-sensory stimuli, embodied sport practices, and neo-tribal culture values?

How can a sports ecosphere be attuned with environmental integrity?

How can attunement ground interventions for collective action to reimagine the sport-nature relationship and build alternative regimes of care?

To conclude, the outlined inquiry forms an ambitious research agenda for thoroughly sensitizing the social-ecosystems of outdoor sport environments. It suggests a holistic approach to better understand the sport-nature-culture nexus as well as reimagine the environmental practices and character of outdoor sports. It thus builds foundations for developing an interdisciplinary social sport ecology domain. Engrained in this approach are political processes that influence atmospheres, attitudes, and behaviors of outdoor sports' participants toward the environment. Beginning to address comprehensively the complex dynamics of sports ecospheres that, in turn, determine behaviors, we can also gain insight on the political forces and mechanisms that constrain or facilitate a cultural change for preserving environmental integrity.

## Author's note

Outdoor sports are strongly connected to the natural environment in which they take place, generating multifaceted impacts that need to be managed in order to protect the ecological integrity of those places. Attention on this matter is part of sport ecology, which has emerged as a subdiscipline of sport management. Nonetheless, current perspectives fail to appreciate the complex relationship among sport, environment, social dynamics and culture. Conversely, leisure studies takes on ‘nature sports' help to capture the ways that physical environment articulates an alternative ontology in which nature and culture form ‘co-constitutive' relationships. Building on these premises, we provide a conceptual analysis of participants' embodied interactions with nature and the socio-cultural dynamics that influence them. The aim is to bridge the divide between nature and culture, thereby informing interventions to build more sustainable, social-ecosystems of care toward the environment. We thus put forward a comprehensive framework for managing outdoor sportscapes as socio-ecological systems. The contribution of this framework progresses the field of sport ecology by offering a holistic approach to better understand and reimagine the environmental practices and character of outdoor sports. Therefore, it advances the discourse on sport ecology by building a sensitized social-ecosystems approach of outdoor sport environments.

## Author contributions

Both authors listed have made a substantial, direct and intellectual contribution to the work, and approved it for publication.

## Funding

Partial funding for open access to this research was provided by University of Tennessee's Open Publishing Support Fund.

## Conflict of interest

Author VZ was employed by Leisure Insights Consultancy, Leeds, United Kingdom. The remaining author declares that the research was conducted in the absence of any commercial or financial relationships that could be construed as a potential conflict of interest.

## Publisher's note

All claims expressed in this article are solely those of the authors and do not necessarily represent those of their affiliated organizations, or those of the publisher, the editors and the reviewers. Any product that may be evaluated in this article, or claim that may be made by its manufacturer, is not guaranteed or endorsed by the publisher.
